# Establishment and evaluation of ectopic and orthotopic prostate cancer models using cell sheet technology

**DOI:** 10.1186/s12967-022-03575-5

**Published:** 2022-08-29

**Authors:** Dongliang Zhang, Ying Wang, Lei Liu, Zeng Li, Shengke Yang, Weixin Zhao, Xiang Wang, Hong Liao, Shukui Zhou

**Affiliations:** 1 Department of Urology, Sichuan Cancer Hospital & Institute, Sichuan Cancer Center, School of Medicine, University of Electronic Science and Technology of China, Chengdu, 610041 China; 2 Department of Urology, Shanghai General Hospital, Shanghai Jiao Tong University School of Medicine, Shanghai, China; 3 Department of Urology, Shanghai Jiao Tong University Affiliated Sixth People’s Hospital, Shanghai Eastern Institute of Urologic Reconstruction, Shanghai Jiao Tong University, Shanghai, 200233 China; 4 Wake Forest Institute for Regenerative Medicine, Wake Forest University School of Medicine, Winston Salem, NC 27157 USA

**Keywords:** Cell sheet technology, Prostate cancer model, Ectopic transplantation, Orthotopic transplantation, Tumor microenvironment

## Abstract

**Background:**

The traditional prostate cancer (PCa) model is established by injecting cell suspension and is associated with a low tumor formation rate. Cell sheet technology is one of the advancements in tissue engineering for 3D cell-based therapy. In this study, we established ectopic and orthotopic PCa models by cell sheet technology, and then compared the efficiency of tumor formation with cell suspension injection.

**Methods:**

DU145 cells were seeded on 35 mm temperature-sensitive dishes to form PCa cell sheets, while the cell suspension with the same cell density was prepared. After transplanting into the nude mice, the tumor volumes were measured every 3 days and the tumor growth curves were conducted. At the time points of 2 weeks and 4 weeks after the transplantation, magnetic resonance imaging (MRI) was used to evaluate the transplanting site and distant metastasis. Finally, the mice were sacrificed, and the related tissues were harvested for the further histological evaluation.

**Results:**

The orthotopic tumor formation rate of the cell sheet injection group was obviously better than that in cell suspension injection group (100% vs 67%). Compared with cell suspension injection, the tumors of DU145 cell sheet fragments injection had the higher density of micro-vessels, more collagen deposition, and lower apoptosis rate. There was no evidence of metastasis in forelimb, lung and liver was found by MRI and histological tests.

**Conclusion:**

We successfully cultured the DU145 cell sheet and can be used to establish ectopic and orthotopic PCa tumor-bearing models, which provide an application potential for preclinical drug development, drug-resistance mechanisms and patient individualized therapy.

## Introduction

Globally, Prostate Cancer (PCa) is the second most common cancer in men, accounting for 13.5%, second only to lung Cancer, with the highest incidence of male tumors in Europe and the USA [1]. The new cases of prostate cancer is stabilized in most countries except for a rapid growth trend at 2.6%/year in China [2], where it has become the most common male urinary tumors. Due to the widespread development of early screening, localized PCa accounted for 78%, and 5-year relative survival rates have reached 98% in the USA [3]. However, compared with no distant metastasis, 5-year relative survival rate dropped from 99% to 31% in PCa patients with metastasis [3].

Most primary PCa inevitably progresses into castration-resistant prostate cancer (CRPC) after androgen deprivation therapy. The CRPC is considered as the final stage of the disease with limited therapeutic options to date. However, the relevant resistance mechanisms are still unclear, including androgen receptor-related signaling pathways, androgens synthesis, lineage plasticity and phenotype switching, gene polymorphisms, etc. [4, 5]. Tumor research relies on accurate animal models. One of the major causes for slowing down the development of novel drugs and revelation of resistance mechanisms is the lack of suitable animal models to accurately simulate the growth, metastasis, and drug resistance of PCa in patients.

Previously, many scholars have reported the successful establishment of animal model of PCa by cell injection [6–8]. However, the cell suspension injection method has some limitations. First, enzyme digestion is required to obtain tumor cell suspension, which will lose a considerable number of cells, destroy the extracellular matrix (ECM) components and affect cell activity, resulting in reduced transplantation efficiency after cell injection, and that is associated with low survival rate and unstable tumor formation rate [9]. Secondly, to control the error in drug or clinical study, the size of the transplanted tumor should be uniform, but the growth rate and grafted tumor volume are not well controlled by cell suspension injection. Furthermore, the ECM and various cytokines are the main components of tumor cell microenvironment [10]. ECM is usually broken down and lost during tryptic digestion, which inevitably affects the transmission of signaling pathways, resulting in changes of gene/protein expression and phenotypic characteristics of cancer cells. Due to this discrepancy, it is difficult to translate the results of tumor research in the lab into clinical application. It has been suggested that only 20–25% of high-profile cancer studies can be replicated by an industrial laboratory [11], and there are also frequent differences between large-scale drug screenings of cancer cell lines [12]. Thus, the quality of animal models must be improved to accurately evaluate the effectiveness of antitumor drugs or novel targeted therapies.

In recent years, the development of tumor cell sheet technology is expected to overcome the above shortcomings. Cell sheets are harvested using a continuous culture method and a physical approach. Avoiding the trypsin digestion and additional scaffold materials, cells can be harvested together with endogenous ECM, cell–matrix and cell–cell contacts [13]. Cell sheets are composed of cells, ECM and cytokine, and their structure is similar to natural organization. Our previous study confirmed that the growth factors were abundant in native cell sheets, including significant amounts of transforming growth factor-β, basic fibroblast growth factor and vascular endothelial growth factor [14]. Currently, the cell sheet engineering has already been applied in various organ repair and reconstruction, including heart [15], bladder [16], periodontal ligament [17], skin [18] and urethra [19], etc.

To our knowledge, there are no published studies of PCa models using cell sheet technology. In this study, we established ectopic and orthotopic nude mouse models of PCa using cell sheet technology, and evaluated tumor formation rate, speed, tumor size, marker expression, tumor invasiveness and MRI in vivo imaging compared with using cell suspension as the control. Our study might provide a new animal models of PCa with more clinically relevant potential.

## Materials and methods

### Materials

The cell culture reagents and products, such as the 35 mm temperature-responsive cell culture dishes, were purchased from Thermo Fisher Scientific (Rockford, IL, USA). Male nude mice (age 6 weeks) were provided by Shanghai SLAC Laboratory Animal Co., Ltd. and maintained in a barrier facility on high efficiency particulate air-filtered racks with 12 h dark–light cycles and allowed ad libitum access to food and water. All experimental protocols were approved by a named institutional review board and/or ethical licensing committee. Animal experiments were carried out according to the experimental procedures approved by the Committee for Animal Research of Shanghai Jiaotong University and followed the guidelines for the Care and Use of Laboratory Animals.

### Cell culture and cell sheets harvesting

Human prostate carcinoma cell line DU145 (American Type Culture Collection, Manassas, VA, USA) were cultured in RPMI-1640 medium supplemented with 10% (v/v) fetal bovine serum and 100 U/ml penicillin and 100 μg/ml streptomycin at 37 °C with 5% CO_2_. The medium was changed every 2–3 days. When the cells reached 95% confluence, they were digested with 0.5% trypsin and then dissociated. To improve cell adhesion and cell sheets formation, 35 mm UpCell plates (Thermo Fisher Scientific, USA) were coated with 500 μg/ml rat tail collagen I for 24 h at 4 °C. DU145 cells (10^6^/dish) were cultured at 37 °C in a humidified atmosphere with 5% CO_2_. After 4 days, when the cells reached over-confluency (> 100%), they were harvested as a contiguous cell sheet by holding the culture at 20 °C for 15 min.

### Surface morphology of DU145 cell sheets

Morphological structures of DU145 cell sheets were observed by scanning electron microscopy (SEM, JSM-7800F Prime, JEOL, Japan). Briefly, samples were fixed in 2.5% glutaraldehyde, washed in deionized water, dehydrated in a graded series of ethanol, and dried by lyophilization. The specimens were then sputter coated with platinum and observed with a scanning electron microscope (SU8000 series; HITACHI, Tokyo, Japan).

### Preparation of DU145 cell sheet fragments and cell suspension

After washing with Dulbecco’s phosphate-buffered saline (PBS; Sigma-Aldrich, St. Louis, MO, USA), DU145 cell sheets were chopped into small fragments for syringe injection. The fragment size was smaller than 1 mm × 1 mm so that it could be easily injected through 30 gauge needle. The fragments from a single cell sheet were treated with 0.25% trypsin–EDTA (Sigma-Aldrich, USA) at 37 °C for 10 min and the cell number was counted. After reaching 80–90% confluence, DU145 cells were washed with PBS, followed by a treatment with 0.25% trypsin–EDTA to produce cell suspension. The harvested DU145 cells were washed twice with PBS and then resuspended with BD Matrigel (BD, Bedford, MA, USA).

### Ectopic and orthotopic transplantation by injection

The animals were anesthetized with 2% isoflurane in whole experiment. Twelve male nude mice (age 6 weeks) were randomly assigned to four groups. For ensure the equivalent cell number in different groups. A single cell sheet was harvested and chopped into fragments and the cell number was counted after treated with trypsin–EDTA. And then the equal number of DU145 cell suspension were used as control. For ectopic transplantation, DU145 cell sheet fragments (Group A) and cell suspension (Group B) with equal cell number in 100 μl were subcutaneously injected into the left back of the mice. For orthotopic transplantation, the nude mice were anesthetized and placed in supine position. After the prostate was exposed, 20 μl DU145 cell sheet fragments (Group C) and cell suspension (Group D) were injected into the ventral prostate capsule using a syringe with a 30-gauge needle (50 μl, BD, USA). The abdominal incision was closed using a 5–0 silk suture. Four weeks after injection, the nude mice were sacrificed, and the tumor, left upper limb, liver, lung, bladder, and prostate were harvested for further histological evaluation.

### Tumor measurement

After transplantation, tumor loading was observed and recorded every 3 days. The maximum diameter (a) and minimum diameter (b) of the tumor were measured with micrometer caliper. The tumor volume was assumed to be a semi-ellipsoid and was calculated by the following formula [20]: Tumor volume (V) = a × b^2^ × π/6.

### Magnetic Resonance Imaging (MRI) of the tumor

MRI was used to observe the growth of tumors in vivo and whether there was local invasion or distant metastasis. The nude mice were examined by MRI at the 2nd and 4th week after inoculation and all animals were anesthetized with 2% isoflurane. The MRI was done in 7.0 T small animal MR scanners (Biospec 70/20 USR; Bruker Biospin MRI, Inc., Billerica, MA, USA). T2-weighted MRI was performed by a fast spin-echo sequence, based on specific parameters as follows: TE = 32 ms, TR = 2050 ms, slice thickness = 1 mm, FoV = 30 × 32 mm, and matrix = 256 × 256, scan time≈20 min.

### Histological analysis

For cross-sectional observations, the harvested cell sheets, resected tumors and organs were fixed with 4% paraformaldehyde and embedded in paraffin. The specimens were sliced into 5 μm sections, followed by haematoxylin and eosin (HE) staining and Masson staining. For immunohistochemical staining, the sections were blocked with 1% bovine serum albumin (BSA) and 0.5% Triton-X100, then treated with desmin, vimentin, CK-8, CD31 and type I collagen IgG antibody (rabbit anti-human, 1:500, Abcam, Cambridge, MA, USA) at 4 °C overnight. After washing with PBS, the sections were incubated with a horseradish-peroxidase-conjugated goat anti-rabbit IgG antibody (1:1000; Invitrogen, Carlsbad, CA, USA) for 1 h at room temperature. Finally, the sections were stained with 3,3 N-diaminobenzidine tertrahydrochloride (Sigma-Aldrich, USA) and counterstained with hematoxylin. Images were captured using an upright metallurgical microscope (Olympus, Japan).

### Tumor cell apoptosis analyzed by TUNEL and cleaved caspase-3 immunofluorescence

According to the instructions supplied by the manufacturer, TdT-mediated dUTP nick end labelling (TUNEL) assay was performed with cell apoptosis in situ detection kit (YEASON, China). Briefly, the slides were firstly incubated with proteinase K for 10 min at room temperature, then incubated with Alexa Fluor 488–12-dUTP in TdT buffer for 1 h at 37 °C with protecting from light, and followed by staining with DAPI solution (1 μg/ml, Invitrogen) at room temperature for 5 min. The slides were observed immediately under a fluorescence microscope. Green fluorescence was observed at 520 ± 20 nm with a standard fluorescent filter and DAPI was observed at 460 nm. The cell apoptosis was observed under a fluorescence microscope (Olympus, Japan). In order to detect active caspase-3, cleaved caspase-3 immunofluorescence was used to detect the active (cleaved) form of caspase-3. The resected tumors in each group were fixed with 4% paraformaldehyde and embedded in paraffin. Sections were then permeabilized with 0.5% Triton X-100 (Invitrogen) and blocked with 5% bovine serum albumin for 30 min. Then, sections were incubated with an optimal concentration of rabbit monoclonal anti–cleaved caspase-3 antibody (1:300, Abcam, Cambridge, MA, USA) overnight at 4 °C, then incubated with anti–rabbit secondary antibody (1:500; Alexa Fluor 470; Invitrogen, Carlsbad, CA, USA) for 45 min at 37 °C, followed by washing 3 times with PBS. The cell nucleus was stained with DAPI (1 μg/ml, Invitrogen) for 30 s. The cell apoptosis was observed under a fluorescence microscope (Olympus, Japan).

### Statistical analyses

SPSS version 19 (IBM, USA) was used for statistical analysis. All data are presented as mean ± SD. An unpaired Student’s *t* test was performed to compare differences between two groups. One-way analysis of variance was performed for the comparison of multiple groups. P < 0.05 was considered a significant difference.

## Results

### Culture and identification of DU145 cell sheets

**Fig. 1:**
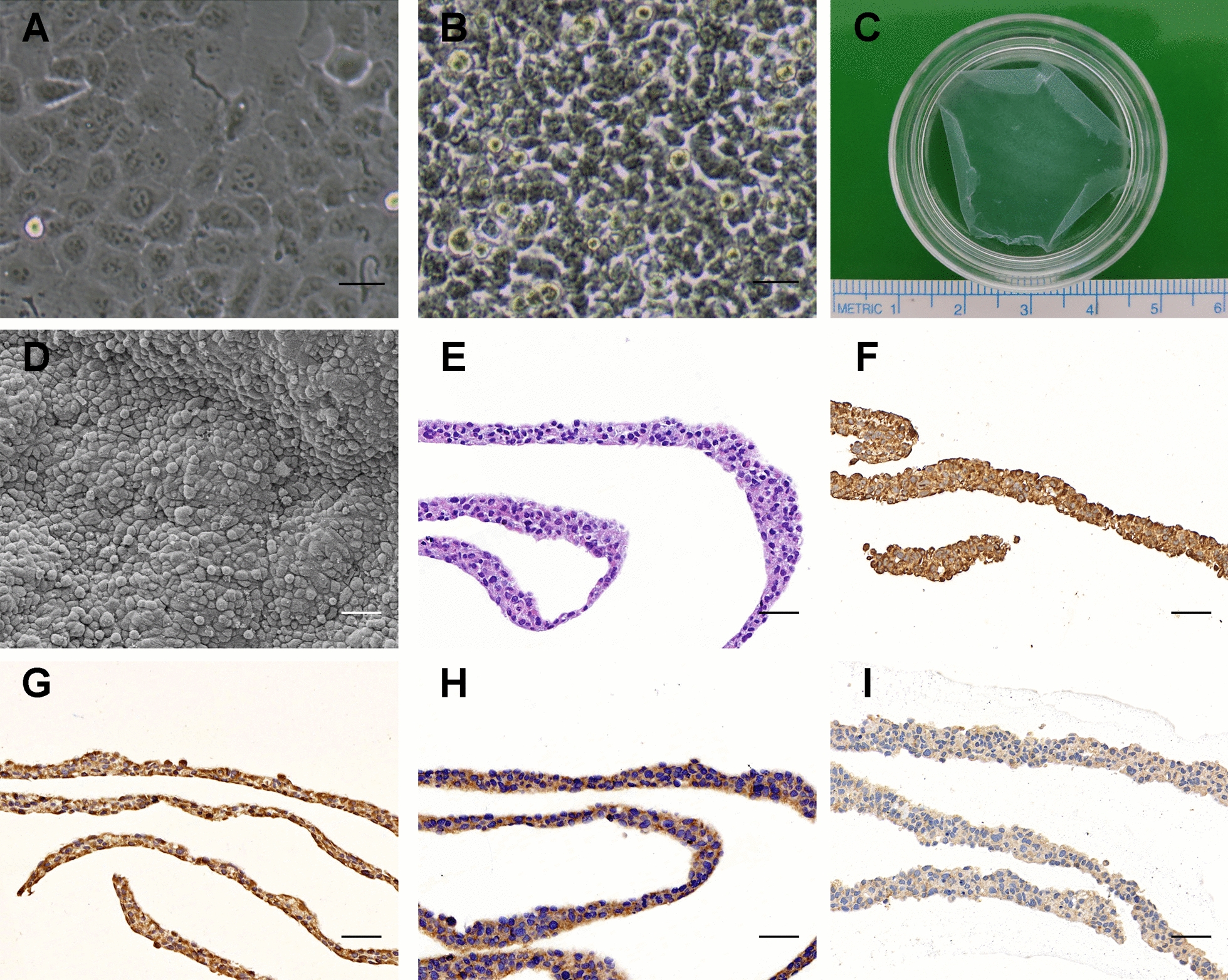
Culture, harvesting and identification of DU145 cell sheets. **A**: DU145 cell culture. Cells reached 90% confluence, manifested as polygonal or spindle shaped. Scale bar: 100 μm. **B**: DU145 cell sheet was observed under light microscopy. Cells were closely connected with white collagen. Scale bar: 100 μm. **C**: DU145 cell sheet in the 35 mm dish. **D**: SEM images of DU145 cell sheets. Scale bar: 100 μm. **E**: HE staining revealed that the DU145 cell sheets were composed of 3–5 layers of cells. **F**–**I**: Immunohistochemical staining was used to identify DU145 cell sheets. **F**: CK-8, **G**: vimentin, **H**: type I collagen, **I**: desmin. Scale bar: **E**–**I**, 50 μm

The PCa cell sheets were cultured in vitro with DU145 cell lines. DU145 cells are polygonal or spindle shaped, with different sizes and clear cellular outline. DU145 cells showed highly proliferative, irregular cell morphology and unclear intercellular boundary in the cell sheets. After lowering the culture temperature, the cell sheets were completely stripped and presented as translucent slices. The SEM scan showed that the surface of DU145 cell sheet was smooth and the cells were tightly connected with abundant fiber cords and collagen. HE staining showed that DU145 cell sheet was composed of 3–5 layers of cells and the thickness of DU145 cell sheet was 32.6 ± 7.5 µm. Vimentin, CK-8 and type I collagen immunohistochemical staining was positive, while desmin was negative (Fig. 1).

### Tumor transplantation and measurement

**Fig. 2:**
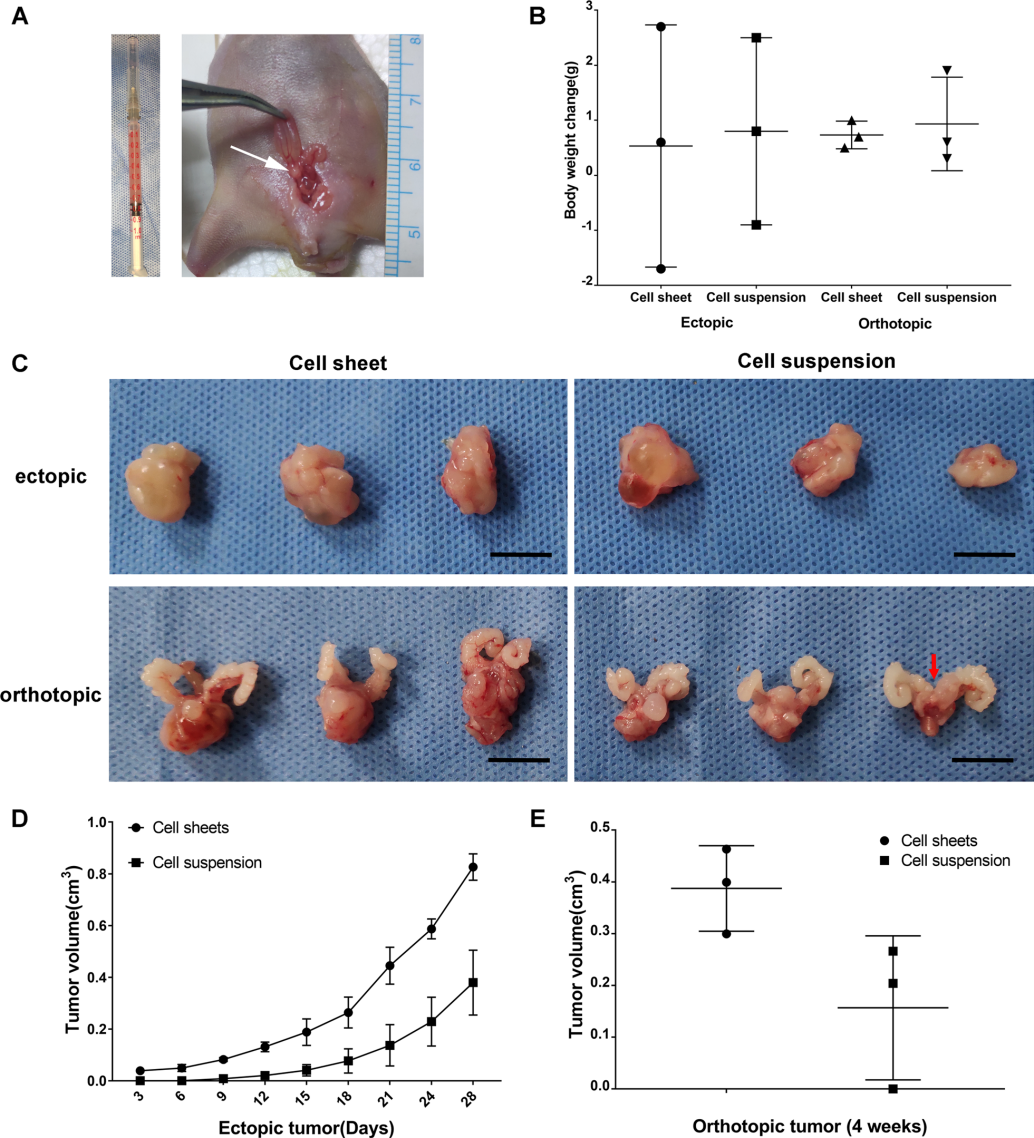
Tumor growth measurement and gross observation. **A**: Cell sheet fragment in the syringe and a satisfactory intracapsular prostatic injection (white arrow). **B**: Body weight changes during observation. **C**: Gross observation of tumors in the four groups. Red arrow shows the prostate with no tumor growing. Scale bar: 1 cm. **D**: Ectopic tumor growth curve. **E**: Orthotopic tumor volumes after 4 weeks

Cell sheet fragments were soft and plastic and easily injected into the prostate capsule with a 30-gauge needle (Fig. 2A). There was no significant change in body weight during the observation period for all group (Fig. 2B). The mice were killed and tumor extracted after 4 weeks transplantation (Fig. 2C). For ectopic transplantation, infiltrative tumor growth was observed in Group A, shown as an irregular solid mass with unclear boundary, which made it difficult to completely remove the surrounding tissue. The tumor in Group B had a complete capsule and was easy to be exfoliated. There was an area of bright and round central necrosis after dissection. For orthotopic transplantation, the tumors in Group C exhibited a large volume of solid mass beneath the seminal vesicles, with irregular morphology, while the volume of solid mass in Group D was significantly smaller. For ectopic transplantation, both of group A (3/3) and group B (3/3) exhibited 100% tumorigenesis rate after 4 weeks transplantation, but tumor growth in Group A was faster than that in Group B (Fig. 2D). Four weeks after transplantation, the tumor volume of Group A and B was 0.83 ± 0.03 and 0.38 ± 0.07 cm^3^, respectively (p < 0.05). For orthotopic transplantation, Group C exhibited 100% tumorigenesis rate, which was higher than that in Group D (66.7%). Four weeks after transplantation, the tumor volume of Groups C and D was 0.38 ± 0.05 and 0.16 ± 0.08 cm^3^. Statistical analysis could not be done for no tumor growth was observed in one case of group D, but the mean tumor volume comparison showed the growth rate of DU145 cell sheet fragments injection was significantly faster than cell suspension injection in situ (Fig. 2E).

### MRI in vivo

**Fig. 3:**
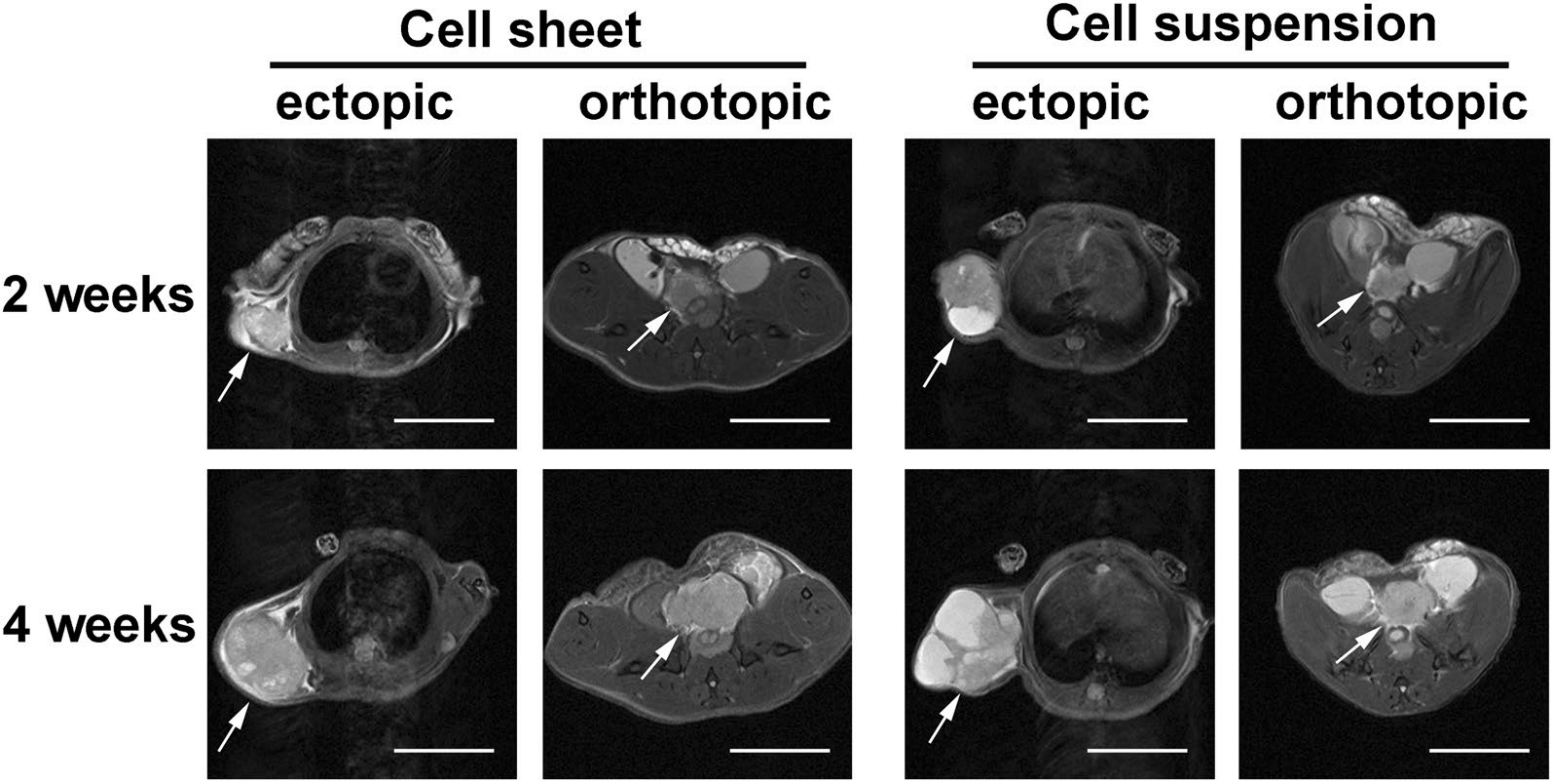
MRI in vivo. MRI was performed at 2 and 4 weeks after transplantation. White arrows represent the area of the tumor. Scale bar: 1 cm

Figure 3 showed T2-weighted MRI images in vivo. All the tumors were detected as hyperintensity areas and uneven signal enhancement on T2-weighted images. Two weeks after subcutaneous injection, the tumors appeared as quasi-circular homogeneous solid masses in Group A, while those in Group B were cystic solid masses with fluid necrosis. Four weeks after subcutaneous injection, the solid tumor was enlarged in the cell sheet group without obvious liquefaction necrosis, while the liquid necrosis area of tumor was enlarged in the cell suspension group and presented as cystic septations. Two weeks after injection, the tumors in Group C and D were located at the front of the rectum, and appeared as irregular solid masses of similar size. Four weeks after injection, the tumor volume increased more significantly in Group C, and even filled the pelvis, while the tumor growth was slow in the Group D.

### Tumor histological analysis

**Fig. 4:**
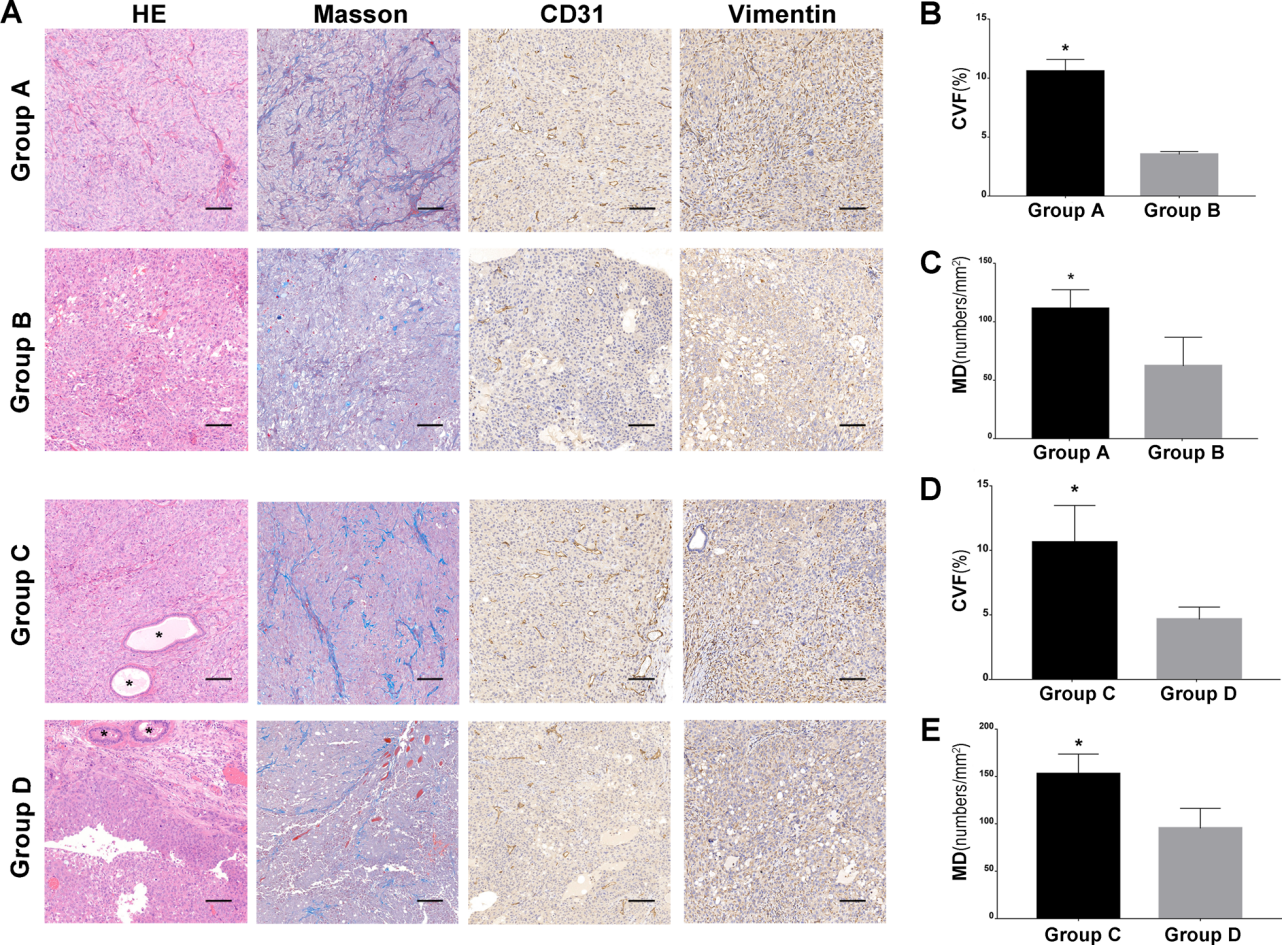
Histological analysis. **A**: Histopathological examination after ectopic and orthotopic transplantation. Dense growth of tumor cells, abundant collagen deposition and high microvessel density were found in Groups A and C compared with Groups B and D. In Group C, a large number of tumor cells grew quickly, leaving a small amount of normal gland tissue. Vimentin was positive in all groups. Scale bar: 100 μm. **B**: Comparison of collagen content in Groups A and B (*p < 0.05). **C**: Comparison of microvessel density in Groups A and B (*p < 0.05). **D**: Comparison of collagen content in Groups C and D (*p < 0.05). E: Comparison of microvessel density in Groups C and D (*p < 0.05). CVF: collagen volume fraction; MD: microvessel density

The cell number per DU145 cell sheet was about 2 × 10^6^ and the equal number of DU145 cells were used for the each group. Four weeks after injection, HE staining of cell sheet fragment injection (Group A and group C) showed dense growth of tumor cells, with no obvious liquefaction necrosis in the center. For cell suspension injection (Group B and Group D), the cell–cell connection was loose, and obvious liquefaction necrosis was observed in the tumor center, accompanied with vacuolation and hyaline changes. Masson staining showed more abundant collagen deposition in Group A and C than that in Group B and D (Fig. 4B and D). The endothelium of small vessel was stained with CD31. Ten nonconsecutive visual field were randomly selected from each group. The average number of vessels per field was expressed as the microvessel density. The median microvessel density of Group A and Group B was 111.2 ± 9.4 and 62.2 ± 14.2/mm^2^ (p < 0.05), respectively. The median microvessel density of Group C and group D was 152.6 ± 12.2 and 95.2 ± 12.2/mm^2^ (p < 0.05), respectively (Fig. 4C and E). Regardless of ectopic or orthotopic transplantation, there were more abundant blood vessels with cell sheet fragment injection than that with cell suspension injection. Immunohistochemical staining of vimentin was positive in ectopic and orthotopic transplantation (Fig. 4A). There was no significant difference in tumor antigen expression.

### Tumor cell apoptosis analysis

**Fig. 5:**
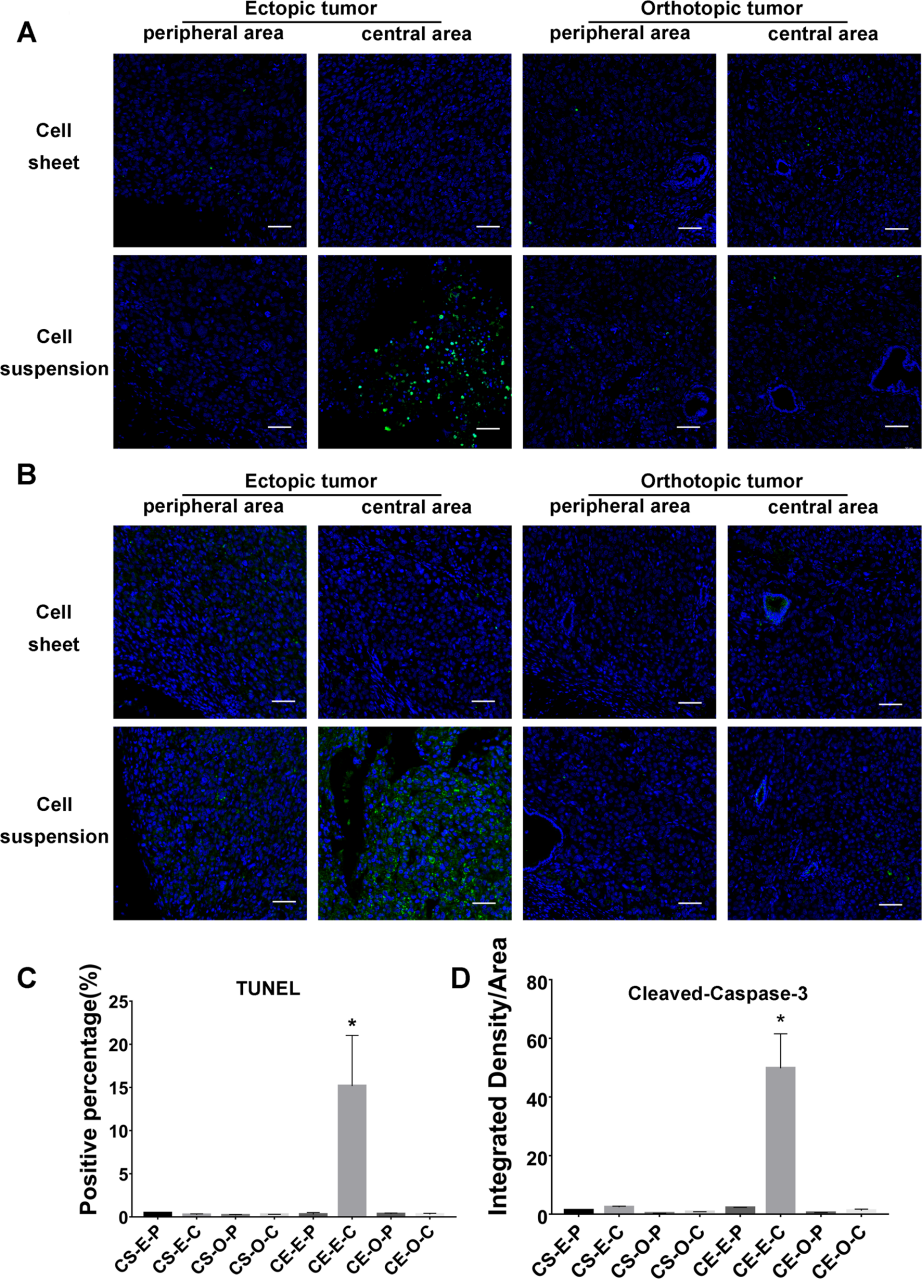
Apoptosis analysis by TUNEL staining and cleaved caspase-3 immunofluorescence staining. Significant numbers of apoptotic cells were observed in the central regions of the tumor in Group B than Group A, while there was no significant difference in apoptosis cells between Groups C and D. **A** TUNEL staining, the apoptotic nuclei were stained green and the nuclei were stained blue with DAPI. **B** Cleaved-Caspase-3 immunofluorescence staining, when the cell was stimulated by apoptosis, the activated Cleaved-Caspase 3 in the cytoplasm was indicated as green. The nuclei were stained blue with DAPI. **C** Histogram of the percentage of TUNEL positive nuclei. **D** Histogram of the integrated density of Cleaved-Caspase 3 positive area. *CS* cell sheet, *CE* cell suspension, *E* ectopic tumor, *O* orthotopic tumor, *P* peripheral area, *C* central area. *: p < 0.05. Scale bar: 50 μm

Four weeks after subcutaneous injection, TUNEL-positive cells were barely detected in the peripheral regions of tumors, which showed no significant differences between Group A and B. In contrast, significant numbers of apoptotic cells were observed in the central regions of the tumor in Group B. Overall, compared to cell sheet fragment injection, the number of TUNEL-positive cells was more than that after cell suspension injection. After in situ injection, in the peripheral or central regions of the tumor, there was no significant difference in TUNEL-positive cells between Groups C and D. The result of cleaved caspase-3 immunofluorescence staining showed the same trend (Fig. 5).

### Pathological analysis of distant metastasis

**Fig. 6:**
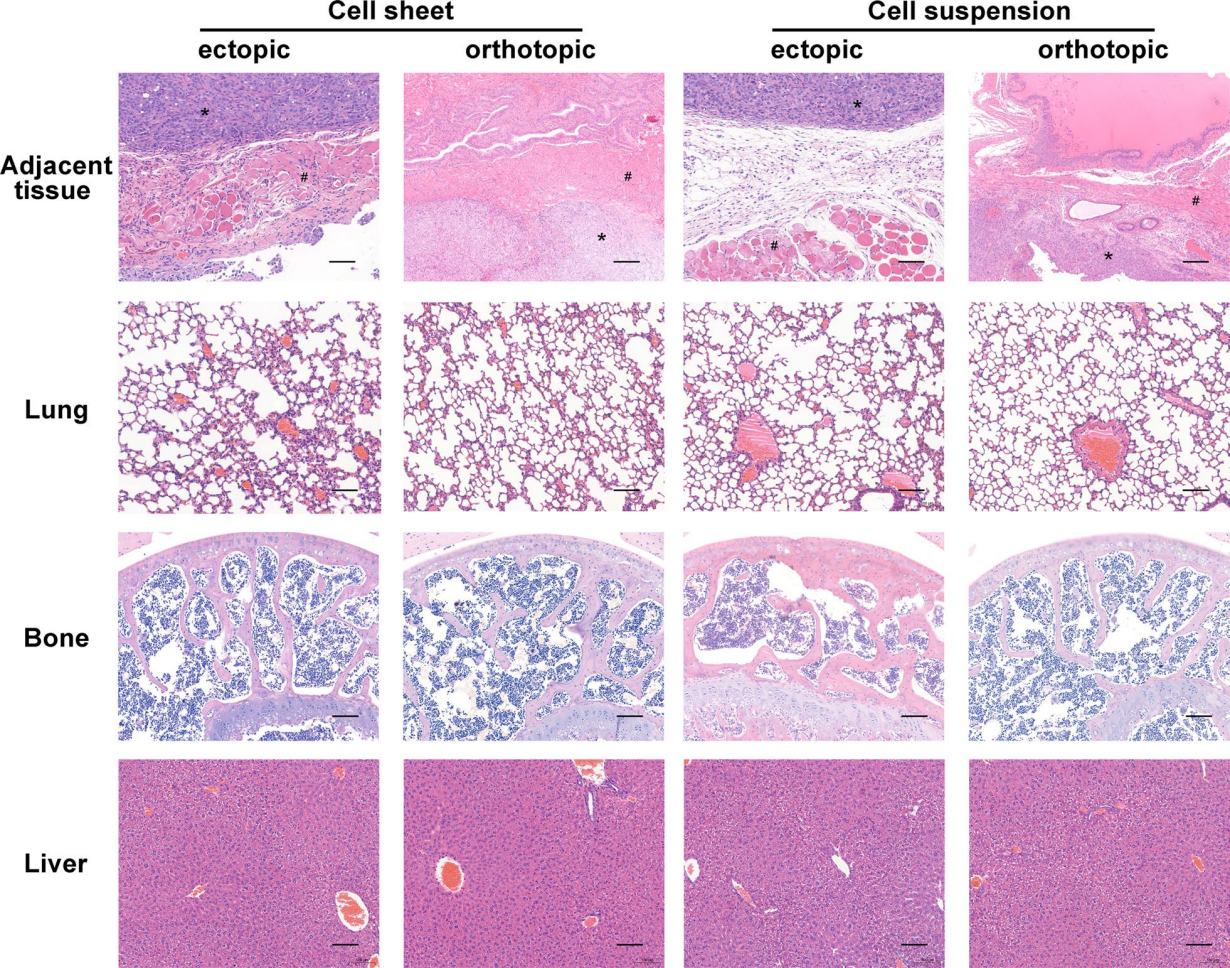
Histopathological examination of the major organs 4 weeks after transplantation. DU145 cell sheet groups had no distant metastasis during the observation period, but they had direct invasion of the surrounding tissues compared with the cell suspension groups. #: adjacent muscle, *: tumor. Scale bar: 100 μm

The HE staining was performed on the major organs (muscle, bladder, lung, liver and bone) after 4 weeks injection (Fig. 6). Four weeks after subcutaneous injection, we observed tumor infiltration into the muscle in the Group A, while the tumor capsule was intact in Group B and no apparent invasion was observed. Tumor invasion of the surrounding bladder muscles was also found in Group C, but not in Group D. There was no obvious metastasis in bone, liver and lung in each group.

## Discussion

Metastatic PCa, especially metastatic CRPC, is still difficult to treat clinically and has poor prognosis. The PCa cell injection method was the conventional approach to establish preclinical animal models. However, this method has some limitations that cannot be overcome. Tissue engineering technology provides a new method for PCa model construction. The three-dimensional (3D) culture has showed a trend to gradually replace the flat culture technique in fields of tissue engineering, such as organ-on-a-chip biosystem [21]. Cell sheets can effectively preserve the ECM components, relevant growth factors, cell growth microenvironment and microstructure, thus cell sheet technology is also a type of 3D cell culture. In this study, we developed the novel ectopic and orthotopic PCa models using cell sheet technology. Compared with conventional cell injection method, the cell sheet-transplanting method improved the engraftment efficiency and enhanced tumor generation in animal model, which provides an ideal tumor model for the research.

PCa is an endocrine-dependent tumor. At present, the cornerstone of advanced metastatic PCa treatment is still endocrine therapy, but after a median 18–24 months of endocrine therapy, almost all patients progress to CRPC [22]. As the first PCa cell lines, DU145 cells, was isolated from brain metastases lesions of a PCa patient, and is an androgen-independent PCa cell line with high vimentin and CK-8 expression and no detectable desmin expression [23, 24]. To prepare an animal model of metastatic CRPC, we selected DU145 cell lines as the seed cells. We found rat tail collagen I coating on the surface of the culture dish was essential to harvest an intact DU145 cell sheet. DU145 cells formed clusters rather than cell sheets without collagen coating. We speculate that, compared with fibroblasts [25] or mesenchymal stem cells [26], DU145 cells have poor adherent and spreading ability, and lack of sufficient collagen secretion, which also results in thinner cell sheets. The PCa is an indolent tumor, have the characteristics of low proliferation and slow growth, which also makes it difficult to form the cell sheet.

Tumor cells can be injected into animal organs in situ to form orthotopic cancer models, which can better simulate the whole process of tumor genesis, development, invasion and metastasis in vivo [27]. However, single cells in cell suspension lack effective intercellular communication and niche, and spontaneously migrate into the blood circulation and surrounding tissue after situ cell injection. It is also difficult to ensure transplanted cells from the cell suspension to stay or distribute evenly in the organ, which result in a low success rate of in situ transplantation. The cell sheets are consists of cells and ECM. Several cytokines and growth factors are combined with the ECM and may thus play an important role in tissue regeneration applications [28]. Our study showed that DU145 cell sheet fragment transplantation can accelerate the tumor formation and greatly improve the efficiency of tumor formation. At the initial stage, compared with cell suspensions injection, the cell sheet based transplantation method improved the engraftment efficiency by 13-fold in mouse subcutaneous tissue [29]. Moreover, the average tumor volume using cell sheet-transplanting method was 10 times larger than that with cell suspension injection on day 14 after transplantation [29]. In recent years, cancer cell sheets have been used successfully to generate tumor-bearing animal models by subcutaneously or in situ injection, such as osteosarcoma [30], hepatocellular carcinoma (HCC) [31], mammary gland adenocarcinoma [29] and lung squamous cell cancer [32]. Alshareeda et al. [31] have developed an HCC sheet and transplanted it into the liver to create a tumor-bearing animal model within a month. The author placed the HCC sheet cover a single lobe of the rat's liver. There are abundant ECM proteins and cell–cell junction proteins in cell sheets. Therefore, the cell sheets can adhere to tissue surfaces without sutures. This direct adhesion method is suitable for smooth and flat organs, but it needs good exposure of the transplantation site, and has a risk of slippage and displacement with the organs moving. The prostate is located deep in the perineum within a narrow space. In our study, the cell sheet fragment was injected into the prostate capsule using a syringe, which is more convenient and accurate, and minimally invasive than the adhesion method.

Angiogenesis is necessary for tumor survival, which helps growing tumors to obtain adequate nutrition and discard metabolic waste. In the previous study, tumors did not exceed a mean diameter of 0.93 ± 0.29 mm during the avascular phase, but after vascularization, tumor volume increased rapidly, and reached a mean diameter of 8.0 ± 2.5 mm by day 7 [33]. As a cytokine repository, various growth factors, cytokines, and chemokines are deposited within the ECMs through binding to specific ECM molecules [34]. The ECM and growth factors are involved in tumor-induced angiogenesis, vascular-stabilizing and maintenance of vessel endothelial cell survival [35, 36]. Moreover, cell-derived ECM can avoid immune and inflammatory reactions after transplantation [37]. In our study, the vascular density in tumor tissues of the cell sheet transplantation group was significantly higher than that of the cell suspension injection group. This can be explained by the preservation of intact ECM ultrastructure and abundant cytokines in the cell sheet fragment. And then, the cell sheet transplanted tumor was supplied with sufficient oxygen and nutrients from the host tissues. The tumor formation rate of cell sheets in situ transplantation reached 100% (3/3), which was significantly higher than that of cell injection (67%, 2/3). Meanwhile, the tumor growth was accelerated significantly after vascularization by cell sheet fragments transplantation, and the tumor infiltrated surrounding connective tissue which has newly formed vessel network. Due to the large subcutaneous space, and higher number of original injected cells, subcutaneous tumors are obviously larger than orthotopic tumor for both cell sheet fragment and cell suspension injections, but the supply of oxygen and nutrients become worse with fast increasing tumor size. Caspase family is a key element in the process of cell apoptosis. Under normal conditions, the cytosolic caspase-3 is inactive and exists as pro-caspase-3 [38]. When cells was stimulated by apoptotic signals, pro-caspase-3 will be transformed into cleaved caspase-3 and further to be activated, and then induce cell apoptosis [39]. The pro-caspase-3 is cleaved only when apoptosis event occurs. Both the TUNEL staining and cleaved caspase-3 immunofluorescence showed more apoptotic cells were observed in the central regions of the tumor in ectopic cell suspension transplantation, while there was no significant difference in orthotopic transplantation. We believe that hypoxia and insufficient blood supply are more likely to occur in the central region of tumor tissue, which led to central apoptosis in Group B, but not in Group A, which had more microvessel formation.

However, this study had a few limitations. First, there is a smaller number of cases in each group, which may lead to statistical error and bias. The reason for choosing small groups is based on the study aims. This is a preliminary and explorative study. Our research design may be suitable for developing a new in vivo model that focuses on the feasibility and efficiency. In addition, the average tumor diameter must be limited in 20 mm in the mouse model for animal ethical protection. Since prostate cancer is an inert tumor with slow growth and progression, the observation time after the tumor transplantation in our study is not long enough to observe palpable metastasis in the body. Lastly, studying the expression of human clinical targets in murine implant tumors is the key to improve the success rate of clinical translation. Though this study have thoroughly compared DU145 cell sheets and suspended cells in tumorigenesis, but we have not compared the traits of the murine tumors with the human clinical targets at the protein and gene level, which is important to understand the similarities and differences in tumor biology.

## Conclusion

Based on our knowledge, this is the first study on the development of ectopic and orthotopic PCa model in nude mice using cell sheet technology. The orthotopic tumor formation rate of the DU145 cell sheet implantation group was obviously better than that in DU145 cell suspension injection group (100% vs 67%). Compared to the cell injection, the tumors induced by DU145 cell sheet transplantation grows rapidly with larger and uniform tumor mass. Additionally, the density of vasculature is significantly higher, and the apoptosis rate was lower in the cell sheet fragment injection group. In conclusion, the DU145 cell sheet fragment injection could generate a novel PCa tumor-bearing mouse model which is better than that by traditional cell suspension injection. This new model might expand our vision with the PCa research in preclinical drug development, drug-resistance mechanisms, and patient individualized therapy.

## Data Availability

The datasets used and/or analysed during the current study are available from.the corresponding author on reasonable request.

## Abbreviations

PCa: Prostate cancer
MRI: Magnetic resonance imaging
CRPC: Castration-resistant prostate cancer
ECM: Extracellular matrix
HE: Haematoxylin and eosin
HCC: Hepatocellular carcinoma
